# Long non-coding RNA MIAT regulates blood tumor barrier permeability by functioning as a competing endogenous RNA

**DOI:** 10.1038/s41419-020-03134-0

**Published:** 2020-10-30

**Authors:** Jiayuan He, Yixue Xue, Qingyuan Wang, Xinxin Zhou, Libo Liu, Tianyuan Zhang, Chao Shang, Jun Ma, Teng Ma

**Affiliations:** 1 Department of Neurobiology, School of Life Sciences, China Medical University, Shenyang, 110122 China; 2 Key Laboratory of Cell Biology, Ministry of Public Health of China, and Key Laboratory of Medical Cell Biology, Ministry of Education of China, China Medical University, Shenyang, 110122 China; 3 Liaoning University of Traditional Chinese Medicine, Shenyang, 110034 China

**Keywords:** Blood-brain barrier, Molecular neuroscience

## Abstract

Blood–tumor barrier (BTB) presents a major obstacle to brain drug delivery. Therefore, it is urgent to enhance BTB permeability for the treatment of glioma. In this study, we demonstrated that MIAT, ZAK, and phosphorylated NF*κ*B-p65 (p-NF*κ*B-p65) were upregulated, while miR-140-3p was downregulated in glioma-exposed endothelial cells (GECs) of BTB compared with those in endothelial cells cocultured with astrocytes (ECs) of blood–brain barrier (BBB). MIAT inhibited miR-140-3p expression, increased the expression of ZAK, enhanced the ratio of p-NF*κ*B-p65:NF*κ*B-p65, and promoted the endothelial leakage of BTB. Our current study revealed that miR-140-3p was complementary to the ZAK 3′untranslated regions (3′-UTR), and luciferase activity of ZAK was inhibited by miR-140-3p in 293T cells. MiR-140-3p silencing resulted in an increase in BTB permeability by targeting ZAK, while overexpression of miR-140-3p had the opposite results in GECs of BTB. Overexpression of ZAK induced an increase in BTB permeability, and this effect was related to ZAK’s ability to mediate phosphorylation of NF*κ*B-p65. Conversely, ZAK silencing get opposite results in GECs of BTB. As a molecular sponge of miR-140-3p, MIAT attenuated its negative regulation of the target gene ZAK by adsorbing miR-140-3p. P-NF*κ*B-p65 as a transcription factor negatively regulated the expression of TJ-associated proteins by means of chip assay and luciferase assay. Single or combined application of MIAT and miR-140-3p effectively promoted antitumor drug doxorubicin (Dox) across BTB to induce apoptosis of glioma cells. In summary, MIAT functioned as a miR-140-3p sponge to regulate the expression of its target gene ZAK, which contribution to phosphorylation of NF*κ*B-p65 was associated with an increase in BTB permeability by down-regulating the expression of TJ associated proteins, thereby promoting Dox delivery across BTB. These results might provide a novel strategy and target for chemotherapy of glioma.

## Introduction

Glioma originating from human neuroectoderm is a most common type of intracranial tumor in central nervous system (CNS) which accounts for more than 50% in the CNS tumor, showing high incidence and low survival rate in clinic. The clinical treatment efficiency of glioma depends on the size, type, grade, and location of the tumor, as well as the patient’s age and overall health. The main treatment methods include surgical resection, radiotherapy, chemotherapy, gene therapy, etc. However, the death rate or recurrence rate of glioma after comprehensive treatment is still unacceptable^1,2^. How to improve the prognosis efficacy of glioma is an urgent problem.

The blood–tumor barrier (BTB) locating between brain tumor cells and microvessels can severely block the entry of macromolecules from the blood into tumors, resulting in poor prognosis and low efficacy of glioma^2,3^. Although the growth of glioma can cause BTB partly damage, making it slightly more permeable than the blood–brain barrier (BBB), BTB is still a major obstacle to the delivery of anticancer drugs into the tumor^4^. Therefore, selective opening of BTB is the basis and prerequisite for the treatment of glioma^2^.

Long non-coding RNA (lncRNA) is usually described as a non-coding transcript of more than 200 bp and involved in the occurrence and development of a variety of tumors^5,6^. LncRNA myocardial infarction (MI) associated transcript (MIAT), a myocardial infarction-related transcript, is identified as a susceptible locus of MI on chromosome 22q12.1, which participates in a variety of physiological and pathological processes^7^. MIAT, as a competing endogenous RNA(ceRNA) of miR-150-5p, regulates occurrence and development of cardiac hypertrophy^8^. Moreover, MAIT has an positive effect on endothelial cells function and diabetic retinopathy by reducing invasion, proliferation, apoptosis, and diabetic-induced retinal neovascularization in endothelial cells through ceRNA action^9–11^. These studies suggest that MIAT may be associated with the function of ECs.

MicroRNAs (miRNAs) are noncoding, single-stranded, small RNA molecules with 18–25 nucleotides in length that can inhibit gene expression at the post-transcriptional level through nucleotide base pairings between complementary seed sequences of miRNAs and the target gene 3′untranslated regions (3′-UTR)^12^. Some miRNAs are considered to be proto-oncogenes and tumor suppressor genes, which are used as novel and promising therapeutic targets for cancer treatment by negatively regulating transcription or translation of target gene^1,2^. Some studies have found that miRNA is involved in the regulation of BTB permeability^13^, suggesting that miRNA is closely related to the function of endothelial cells. It has been reported that miR-140-3p is downregulated in many tissue cancers^12^. Similary, in our present application of miRNA microarray analysis technology, we found that the expression of miR-140-3p in the BTB group was significantly downregulated, suggesting that miR-140-3p may be related to the change of BTB permeability. However, the miR-140-3p exact expression, the molecular pathways regulated by miR-140-3p and it influence on BTB permeability are not yet known.

ZO-1-associated kinase (ZAK), a serine–threonine kinase and an upstream of MAPK cascade, locates on chromosome 2q31.1 and encodes sterile alpha motif and leucine zipper^14^. ZAK is identified as having TGFBR1 kinase activity in siRNA screening of human kinases. Phosphorylation of ZAK downstream signaling molecules JNK and p38 plays a role in regulating cardiac hypertrophy^15–17^. It has been reported that ZAK expression is upregulated in process of an increase in intestinal epithelial cell permeability in patients with celiac disease^18^, speculating that ZAK may be involved in the regulation of endothelial cells permeability. However, to the best of our knowledge, ZAK’s roles in modulating tight junction (TJ) of glioma-exposed endothelial cells (GECs) remain to be elucidated. In the present study, the potential functions of ZAK in modulating TJ of GECs are studied.

In recent years, some scholars have proposed that lncRNA and mRNA may play an important regulatory role in the process of life activities through the mutual regulation by using “miRNA-binding sites” as mediators^19,20^. LncRNA can also weak miRNA functions and indirectly regulate the expression of target genes related to miRNAs by the use of ceRNA function in the cytoplasm, except for directly regulating the gene expression in the nucleus^21^. According to the ceRNA score principle, lncRNAs must have at least two or more miRNA response elements (MRE) to compete for miRNA binding and interfere with gene expression as ceRNA^22^. We applied BioEdit to find that MIAT had six MREs completely complementary to miR-140-3p seed regions, suggesting that MIAT may regulate an BTB permeability acting as ceRNA against the function of miR-140-3p.

NF*κ*B-p65 is considered as an important transcription factor belonging to NF*κ*B family and also a key factor controlling gene expression^23^. Phosphorylation of Ser536 on p65 occurs in the cytoplasm, leading to nucleoplasmal transfer and enhanced DNA binding and transcriptional activity of p65^24^. Some researchers have found that TNF-α disrupts the barrier function by mediating the reduction of TJ-associated proteins in endothelial cells of BBB via the NF*κ*B signaling pathway, but the specific mechanism is unknown^25^. The team members hypothesize that ZAK, as a serine–threonine kinase, may phosphorylate Ser536 site on p65, thereby causing changes in BTB permeability.

In the present study, we try to provide a novel strategy for glioma therapy basing on the understanding the interaction between MIAT, miR-140-3p, ZAK, and NF*κ*B-p65 factors and BTB permeability. Firstly, the expression and function of these factors in GECs of BTB would be thoroughly characterized and studied. And then, the regulatory mechanisms of ZAK expression and BTB permeability by MIAT as a miR-140-3p ceRNA would be analyzed. Finally, the regulatory relationships among these factors and their effects on the BTB permeability would further be investigated.

## Materials and methods

### Cell lines and cell culture

Human brain microvascular endothelial cell line hCMEC/D3 was donated by Dr. Couraud (Cochin Institute in Paris). Human U87 glioma cells, human brain normal astrocytes (NHA) and human embryonic kidney cell line HEK-293T cells were purchased from the cell bank of the Chinese Academy of Sciences in Shanghai. HCMEC/D3 cells were cultured in EBM-2 (Lonza company, USA) culture medium for endothelial cells with fetal bovine serum, 5% fetal bovine serum (PAA Laboratories, Australia), 1% cyano-streptomycin, 1% lipid concentrate (Life Technologies, USA), 1 ng/ml bFGF, 1.4 microns of cortisol, 5 microns of ascorbic acid (Sigma-Aldrich, St. Louis, MO, USA), and 10 mM HEPES (PAA Laboratories, Australia). When placed in an incubator at 37 °C, 5% CO_2_ and 100% humidity, the medium was changed every 2 days, and the endothelial cells could grow to a monolayer in about 5–7 days. hCMEC/D3 were limited from 30 to 40 passages. U87 cells were cultured with Dulbecco’s Modified Eagle Medium (Thermo fisher, USA) culture medium containing 10% calf serum, 100 U/ml penicillin and 100 µg/ml streptomycin (Life Technologies, USA). NHA cells were cultured in astrocyte medium RPMI-1640 (GIBCO, Carlsbad, CA, USA). All cells were maintained at 37 °C in a humidified incubator of 5% CO_2_.

### Establishment of in vitro BTB and BBB model

HCMEC/D3 cells were cultured in the upper chamber of the transwell chamber and placed in a six-well plate. At the same time, human glial cells and human glioma U87 cells were plated at a density of 2 × 10^4^ cells/ml. Inside the holes of the other six-well plates. When the endothelial cells were fused to 80% in the small chamber, the transwell chambers inoculated with endothelial cells were transferred to the wells of a six-well plate containing human astrocytes and human glioma U87 cells, respectively. These cells are called ECs (endothelial cells co-cultured with astrocytes) and GECs. The culture medium was administered with 1 ml of culture medium, and 2 ml of the culture medium was administered to the wells of a six-well plate. The new culture medium was replaced for 4 days. All cells were cultured in an incubator containing 5% CO_2_ and 95% air at 37 °C.

### MiRNA expression profiles, qRT-PCR assay, and miRNA target analyses

TaqMan MicroRNA Assay Human Set (Applied Biosystems, Foster, VA, USA) was applied to evaluate the miRNA expression profiles according to our previous studies^13^. RNA in cells was extracted by the Trizol (Life Technologies, USA) method, and the purity and concentration of RNA were detected by UV spectrophotometer. The expression of MIAT and ZAK was detected by PrimeScript RT reagent Kit with gDNA (Perfect Real Time) and SYBR Premix Ex Taq II (TaKaRa, China). The expression of miR-140-3p was detected using the Mir-X miRNA First-Strand Synthesis Kit and TB Premix Ex Taq II (TaKaRa, China), with U6 as the reference gene. The primers and probes used in this study were shown in Supplementary Table S1.

### Cell transfection

Short-hairpin RNA (shRNA) direct against human MIAT, miR-140-3p, ZAK, NF*κ*B-p65 gene, as well as their nontargeting sequences were reconstructed in pGPU6/GFP/Neo vector. The full length MIAT, miR-140-3p, ZAK or NF*κ*B-p65 gene were constructed in pcDNA3.0 or pIRES2-EGFP. And the empty vectors were used as NCs. Cells were seeded in a 24-well plate. We used Lipofectamine 3000 reagent and Opti-MEM I to transfect cells with the plasmids according to the manufacturer’s instructions. G418 was used to select the stable transfected cells. For the transient transfection assays, agomir-140-3p, antagomir-140-3p, and their NC sequence were synthesized. Cells were collected 48 h after transfection.

Reporter vectors construction and luciferase assays HEK-293T cells were seeded in a 96-well plate. MIAT sequence and ZAK-3′UTR sequence were amplified as well as their mutant sequences of miR-140-3p binding sites. Similarly, ZO-1-5′UTR, occludin-5′UTR and claudin-5-5′UTR were amplified by PCR and cloned into the pmirGLO Dual Luciferase Expression Vector (Promega, Madison, WI, USA) to construct wild-type and mutation-type luciferase reporter vectors (Generay Biotech Co., Shanghai, China). Then we, respectively, co-transfected the HEK-293T cells with wild-type pmirGLO-MIAT, mutant-type pmirGLO MIAT (or ZAK-3′UTR-Wt, ZAK-3′UTR-Mut) reporter plasmid and agomir-140-3p. Again, HEK 293T cells were co-transfected with the above TJ-associated proteins wild-type and mutation-type luciferase reporter vectors and the NFκB-p65(+) or NFκB-p65(+) NC using Lipofectamine 3000. The luciferase activities can be calculated after performed with the Dual-Lucifer Reporter Assay System according to the manufacturer’s instructions. The sequences of all sh-RNA templates were shown in Supplementary Table S2. To avoid shRNA-mediated off-target effects, we transfected silencing plasmids at multiple sites and tested transfection effificiency. Then, the shRNA with the best silencing efficiency was selected for functional study. The transfection efficiencies were shown in Supplementary Fig. S1.

### Transendothelial electric resistance (TEER) assay

After in vitro BTB model is set up, a Millicell-ERS (Millipore, Billerica, MA, USA) instrument was applied to detect the TEER value according to our previous literature^13^. The electrical resistance of GEC monolayers cultured on transwell filters was measured using a Millicell-ERS instrument. Electrical resistance was expressed in units of Ω·cm^2^ using the surface area of the transwell insert.

### Horseradism peroxidase (HRP) flux assay

After the establishment of the in vitro BTB model, a serum-free EBM-2 medium containing 0.5 µmol/l HRP was added to the transwell chamber of the in vitro BTB model. Twenty-four hour later, the culture solution in the subchamber of the BTB model was collected, HRP content was measured with an enzyme label, HRP standard curve was drawn with HRP standard, and the HRP amount infiltrated into the subchamber was calculated. HRP = the number of picomoles of HRP per square centimeter surface area per hour.

### Western blot assay

The protein extraction and quantification were applied according to our previous literature^13^. The protein concentration was determined by the bicinchoninic acid (Beyotime Institute of Biotechnology, China) method. The protein samples were separated by SDS-PAGE (Beyotime Institute of Biotechnology, China) electrophoresis. The film was transferred under the condition of voltage 100 V, current 120 mA, 90–120 min. It was then sealed in a sealant (5% skim milk) for 2 h. TTBS was washed off the sealing fluid, and the primary antibody was diluted with primary antibody diluen: ZAK (1:500; abcam, USA), p-NF*κ*B-p65 (1:1000; Thermo Scientific, Beijing, China), NF*κ*B-p65 (1 µg/ml; Thermo Scientific, Beijing, China), ZO-1 (1:300; Thermo Scientific, Beijing, China), occludin (1:200; Thermo Scientific, Beijing, China), and claudin-5 (1:500; Thermo Scientific, Beijing, China) in a certain proportion and sealed with a film at 4 °C overnight. After washing TTBS for three times, add the corresponding secondary antibody and incubate at room temperature for 2 h. After TTBS washing for three times, ECL (Beyotime Institute of Biotechnology, China) was used to glow, take photos, and quantity one software was used for quantitative analysis.

### Immunofluorescence assay

Endothelial cells were seeded at a density of 2000/cm^2^ on a 1.5% gelatin-coated cover slide. After 90% fusion, phosphate-buffered saline (PBS) was washed three times for 5 min each time and fixed with 4% paraformaldehyde for 30 min. PBS was washed three times, 5 min each time, and closed with 5% bovine serum albumin for 15 min, then incubated with corresponding antibodies overnight. PBS was washed three times for 5 min each time, then the cy3-labeled goat anti-rabbit fluorescence secondary antibody was incubated in dark for 30 min, and the nuclei were diluted and stained with DAPI at 1:500 for 10 min. PBS wash three times every 5 min and 50% glycerol sealing piece and in being future-proof BX60 Upright Fluorescence system.

### Reporter vectors construction and dual-luciferase reporter assay

The potential binding sequence and the corresponding mutant sequence of miR-140-3p in MIAT, were amplified by PCR and cloned into the pmirGLO DualLuciferase miRNA Target Expression Vector (Promega, Madison, WI, USA) to construct wild-type and mutation-type luciferase reporter vectors (Generay Biotech Co., Shanghai, China). The biological software Targetscan program (http://www.targetscan.org/) predicted the possible target genes and binding sequences of miR-140-3p, chemically synthesized the wild-type ZAK-WT and mutant ZAK-MUT, and cloned the dual luciferase reporter plasmid containing the pmirGLO promoter. Human embryonic kidney cells HEK-293T were inoculated in 96-well plates. After 24 h, when the cell density was around 60–80%, the cells were co-transfected with wild-type or mutant reporter plasmids and agomir or antigomir simulators. After 48 h, luciferase activity was detected according to solution A and solution B in the luciferase detection kit (Promega, USA), and the fluorescence value of firefly was finally used as the relative luciferase activity of each group.

### RNA immunoprecipitation

The EZMagna RIP Kit (Millipore) was applied according to the manufacturer’s protocol. Complete RNA immunoprecipitation (RIP) lysis buffer was used to lyse GECs cells. Magnetic beads conjugated with anti-argonaute 2 (AGO2) or control anti-immunoglobulin G (IgG) antibody were used to incubate the cell extract. The cell extract was incubated for 6 h at 4 °C. Then, as the protein beads were removed. RT-qPCR analysis was conducted for the purification of RNA. The primers for quantitative reverse transcription polymerase chain reaction (qRT-PCR) were performed listed in Supplementary Table S1, and the sequences of wild-type and mutant plasmids were listed in Supplementary Table S3.

### Chromatin immunoprecipitation (ChIP) assay

The endothelial cells in the growth stage were taken in pairs, and 1% formaldehyde was added into the 100 mm cell culture plate. The cells were cross-linked with the endothelial cells for 10 min at room temperature, and glycine was added and placed at room temperature for 5 min. The cells were buffer lysed with lysate containing PMSF and incubated with micrococcal nuclease for 20 min. After detection of chromatin DNA enzymatic digestion, appropriate amount was taken as the positive control of 2% Input. The remaining solution was added with 5 μl of p-NF*κ*B-p65 antibody (Thermo Scientific, Beijing, China) as the experimental group and 1 mu of normal rabbit IgG as the negative control group, and incubated overnight at 4 °C. DNA cross-linking solution was purified by adding NaCl and protease K. According to the reaction conditions, PCR was performed to verify the binding of NF*κ*B-p65 with ZO-1, occludin, and claudin-5. PCR products were isolated from 3% agarose gel. Primers for chip PCR were shown in Supplementary Table S4.

### Apoptosis detection experiment

After BTB model was successfully constructed, 10 M doxorubicin (Dox) (Beyotime Institute of Biotechnology, Jiangsu, China) was added to the upper chamber of transwell. After 12 h, Annexin V-PE/7-AAD staining detection kit was used to detect the U87 glioma cells apoptosis rate according to the instructions. The U87 glioma cells in the lower chamber were collected and washed with 4 °C PBS and centrifuged twice. The U87 glioma cells in the lower chamber were resuspended with Annexin V binding buffer and stained with Annexin V-PE/7-AAD in dark for 15 min. Then the apoptosis score was obtained by FACSan flow cytometry and analyzed by CELL Quest 3.0 software.

### Statistical analysis

Statistical analysis of the data in the study was completed by SPSS19.0 statistical software. The statistical analysis method was as follows: Data were presented in the form of the mean ± SD, the comparison between the two groups was conducted by *t* test, and the comparison between multiple groups was analyzed by one-way ANOVA and corrected by Bonferroni post-test. *P* < 0.05 was considered statistically significant.

## Result

### MIAT was upregulated in GECs, and MIAT increased BTB permeability

**Fig. 1:**
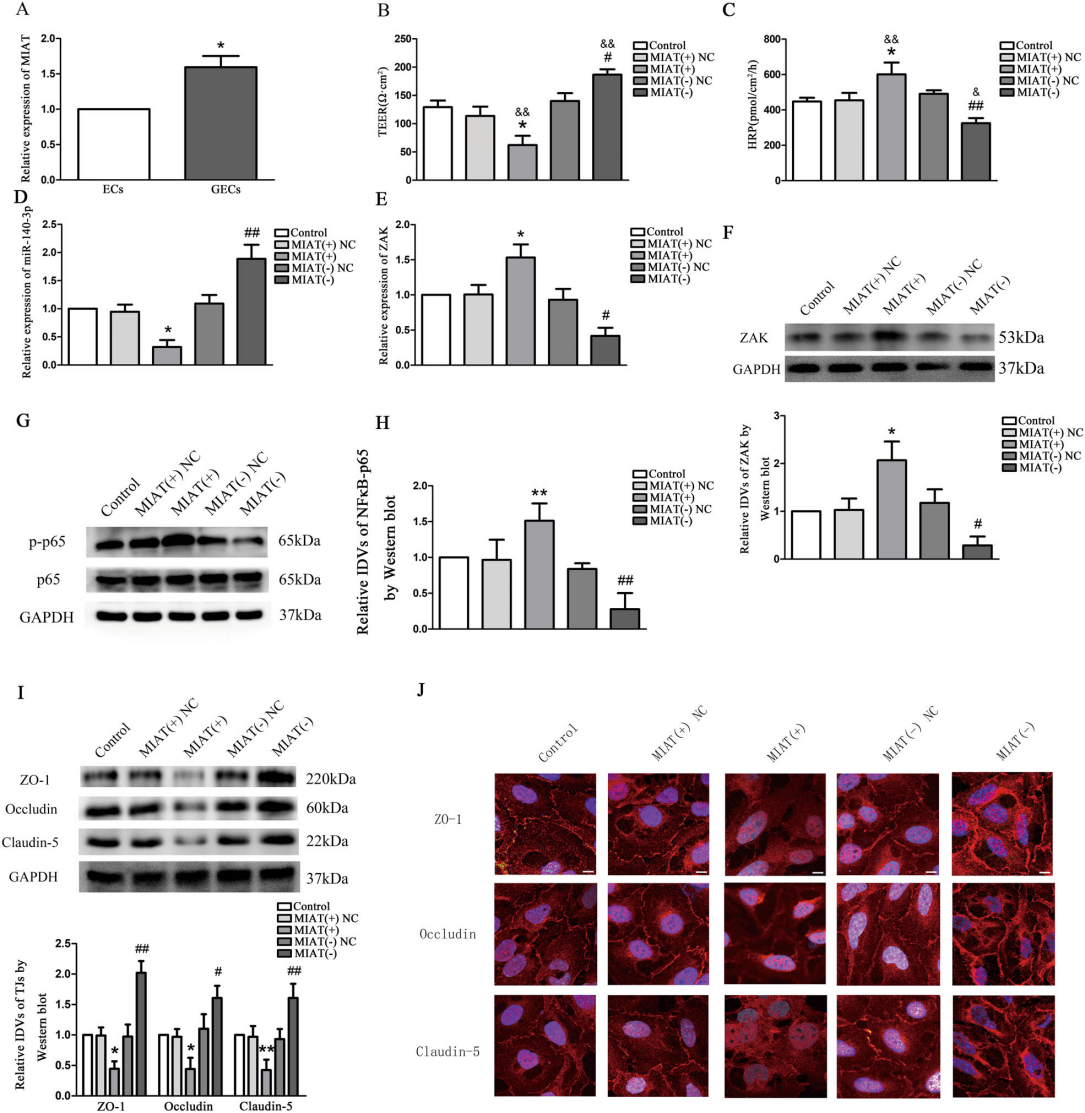
The endogenous expression of MIAT, and effect of MIAT on BTB permeability were detected in vitro. **a** Relative expression of MIAT in ECs and GECs were detected by qRT-PCR. GAPDH was used as an endogenous control. Data represent means ± SD (*n* = 3, each group). *P < 0.05 vs. ECs group. **b** TEER values of GECs were expressed as Ω cm^2^. Data represent mean ± SD (*n* = 3, each group). *P < 0.05 vs. MIAT(+) NC group. ^#^*P* < 0.05 vs. MIAT(−) NC group. ^&&^*P* < 0.01 vs. Control. **c** HRP flux was calculated as pmol/cm^2^/h. Data represent mean ± SD (*n* = 3, each group). *P < 0.05 vs. MIAT(+) NC group. ^##^*P* < 0.01 vs. MIAT(−) NC group. ^&^*P* < 0.05 and ^&&^*P* < 0.01 vs. Control. **d**, **e** The relative expression level of miR-140-3p and ZAK in GECs was mesured by qRT-PCR. U6 RNA level was used as an internal control for miR-140-3p, and GAPDH was used as an endogenous control for ZAK and NF*κ*B-p65. Data represent mean ± SD (*n* = 3, each group). *P < 0.05 vs. MIAT(+) NC group. ^#^*P* < 0.05 and ^##^*P* < 0.01 vs. MIAT(−) NC group. **f** The protein expression of ZAK was detected by western blot. **g**, **h** The protein expression of NF*κ*B-p65 was shown by western blot. **i** Western blot analysis of TJ-associated proteins ZO-1, occludin, and claudin-5 in GECs. For panels **f**–**i**, GAPDH protein levels were used as an endogenous control. Data represent mean ± SD (*n* = 3, each group). *P < 0.05 and **P < 0.01 vs. MIAT(+) NC group. ^#^*P* < 0.05 and ^##^*P* < 0.01 vs. MIAT(−) NC group. **j** Immunofluorescent localization of ZO-1, occludin, and claudin-5 in GECs. ZO-1 (red), occludin ^(^red), and claudin-5 (red) were, respectively, labeled with fluorescent secondary antibody and nuclei were labeled with DAPI. Images were representative of five independent experiments. Scale bar = 20 µm.

The relative expression of MIAT in GECs was 1.6-folds than that in ECs by qRT-PCR (Fig. 1a). In GECs, MIAT was overexpressed by stably transfected MIAT plasmids, silenced by RNAi technology. An in vitro BTB model was constructed to measure TEER and HRP flux to evaluate the permeability of BTB. The results were shown in Fig. 1b, c. TEER value in the MIAT(+) group was significantly decreased (*P* < 0.01 and *P* < 0.05) and HRP flux was increased (*P* < 0.01 and *P* < 0.05) compared with the control and MIAT(+) NC groups, respectively. On the contrary, the TEER value was significantly increased (*P* < 0.01 and *P* < 0.05) and HRP flux was decreased (*P* < 0.05 and *P* < 0.01) in the MIAT(−) group compared with the control and MIAT(−) NC groups, respectively. Moreover, there were no significant differences in TEER value and HRP flux among the control, MIAT(+) NC and MIAT(−) NC groups (*P* > 0.05).

In addition, qRT-PCR results (Fig. 1d, e) presented that miR-140-3p expression was decreased in the MIAT(+) group, and ZAK expressions were increased (*P* < 0.05) compared with the MIAT(+) NC group, respectively. Conversely, miR-140-3p expression was increased in the MIAT(−) group (*P* < 0.01), and ZAK expression was decreased (*P* < 0.05) compared with the MIAT(−) NC group, respectively. No statistically significant differences were obtained among control, MIAT(+) NC and MIAT(−) NC groups. Western blot results indicated (Fig. 1f–h) that the expression of ZAK and phosphorylated NF*κ*B-p65 (p-NF*κ*B-p65) in the MIAT(+) group was increased compared with the MIAT(+) NC group (*P* < 0.05), while the expression of non-phosphorylated NF*κ*B-p65 was not significantly different (*P* > 0.05). On the contrary, ZAK expression in the MIAT(−) group was decreased (*P* < 0.05), and p-NFκB-p65 expression was decreased (*P* < 0.01), whereas non-phosphorylated NF*κ*B-p65 expression was not significantly different (*P* > 0.05) compared with the MIAT(−) NC group. No statistically significant differences were present among control, MIAT(+) NC and MIAT(−) NC groups.

TJ is an important structural and functional basis for maintaining the integrity of BBB and BTB. It is located between adjacent endothelial cells of brain capillaries and composed of transmembrane proteins of occludin and claudins, which forms a sealed structure with cytoplasmic protein components (ZOs) and the cytoskeleton of actin. Changes in TJ-associated protein are generally thought to regulate the permealibility of ECs. As shown in Fig. 1i, Western blotting revealed that the expression of ZO-1 (*P* < 0.05), occludin (*P* < 0.05), and claudin-5 (*P* < 0.01) in the MIAT(+) group was reduced compared with the MIAT(+) NC group; whereas ZO-1 (*P* < 0.01), occludin (*P* < 0.05) and claudin-5 (*P* < 0.01) expressions in the MIAT(−) group were elevated compared with the MIAT(−) NC group. Moreover, ZO-1, occludin and claudin-5 expressions in MIAT(+) NC and MIAT(−) NC groups showed no significant difference (*P* > 0.05) compared with the control group. Immunofluorescence assay was applied to further detect the expression and distribution of TJ-associated proteins, and the changes of ZO-1, occludin, and claudin-5 expression as shown in Fig. 1j were consistent with the above results. Immunofluorescent staining results showed that the relatively continuous distributions of ZO-1, occludin, and claudin-5 in the MIAT(+) group almost disappeared on the cell membrane compared with the control and MIAT(+) NC groups, whereas the continuous distributions of ZO-1, occludin, and claudin-5 of GECs in the MIAT(−) group became more linear compared with the control and MIAT(−) NC groups.

### MiR-140-3p was downregulated in GECs, and miR-140-3p decreased BTB permeability

**Fig. 2:**
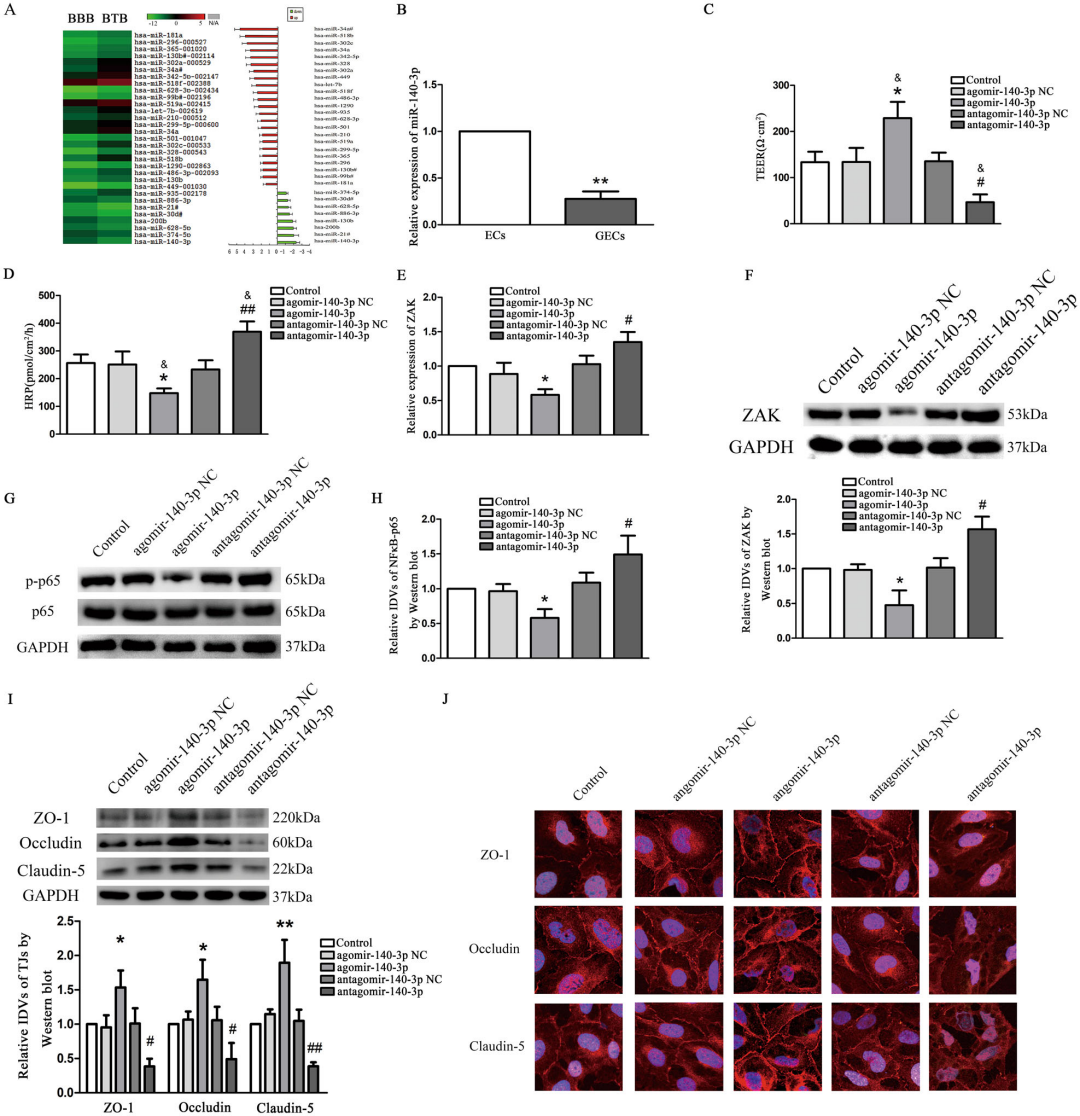
The endogenous expression of miR-140-3p was detected in GECs of BTB and involved in the modulation of BTB permeability in vitro. **a** MiRNA microarray analysis of total RNAs isolated from ECs of BBB and GECs of BTB. Red indicates high relative expression and green indicates low relative expression. **b** Relative expression of miR-140-3p in ECs and GECs were detected by qRT-PCR. U6 RNA level was used as an endogenous control. Data represent mean ± SD (*n* = 3, each group). **P < 0.01 vs. ECs group. **c** MiR-140-3p changed TEER values of GECs. Data represent means ± SD (*n* = 3, each group). *P < 0.05 vs. agomir-140-3p NC group. ^#^*P* < 0.05 vs. antagomir-140-3p NC group. ^&^*P* < 0.05 vs. Control. **d** MiR-140-3p changed HRP flux of GECs. Data represent means ± SD (*n* = 3, each group). *P < 0.05 vs. agomir-140-3p NC group. ^##^*P* < 0.01 vs. antagomir-140-3p NC group. ^&^*P* < 0.05 vs. Control. **e** The relative mRNA expression level of ZAK in GECs was detected by qRT-PCR, and GAPDH was used as an endogenous control. Data represent mean ± SD (*n* = 3, each group). *P < 0.05 vs. agomir-140-3p NC group. ^#^*P* < 0.05 and ^##^*P* < 0.01 vs. antagomir-140-3p NC group. **f** The protein expression of ZAK was shown by western blot. **g**, **h** The protein expression of NF*κ*B-p65 was shown by western blot. **i** Western blot analysis of TJ-associated proteins ZO-1, occludin, and claudin*-*5 in GECs. For panels **f**–**i**, GAPDH protein levels were used as an endogenous control. Data represent mean ± SD (*n* = 3, each group). *P < 0.05 and **P < 0.01 vs. agomir-140-3p NC group. ^#^*P* < 0.05 and ^##^*P* < 0.01 vs. antagomir-140-3p NC group. **j** Immunofluorescent localization of ZO-1, occludin, and claudin-5 in GECs. ZO-1 (red), occludin (red), and claudin-5 (red) were, respectively, labeled with fluorescent secondary antibody and nuclei were labeled with DAPI. Images were representative of five independent experiments. Scale bar = 20 µm.

MiRNA gene expression profiles were successfully obtained from samples in each group. Microarray analysis revealed eight significantly deregulated miRNAs including miR140-3p and 23 significantly upregulated miRNAs in the BTB in comparison with the BBB control group (Fig. 2a). The expression of miR140-3p was significantly downregulated in comparison with BBB control group. This reduction in miR140-3p expression ranked first when considering all deceased miRNAs as determined by miRNA microarray analysis (Fig. 2a). Validated target genes were analyzed using the databases of miRTarBase, MiRDB, and TargetScan. As based upon the results of these analyses, binding of miR140-3p to both ZAK was observed using MiRDB and TargetScan, while use of miRTarBase indicated that there was no binding site between miR140-3p and at the 3′-UTR region of ZAK (Fig. S2a, b).

As shown in Fig. 2b, qRT-PCR results revealed that miR-140-3p expression was reduced by 0.28-folds in GECs compared with ECs group. To further study the function of miR-140-3p in process of regulating BTB permeability, agomir and antagomir of miR-140-3p were transiently transfected into hCMEC/D3, then an in vitro BTB model was constructed by the use of hCMEC/D3 with miR-140-3p overexpression and silencing. The results illustrated that the over-expression of miR-140-3p markedly increased the TEER value of the BTB model in vitro (Fig. 2c) compared with the control and agomir-140-3p NC groups, and decreased the amount of HRP exudation (Fig. 2d) compared with the control and antagomir-140-3p NC groups. No statistically significant differences were obtained among the control, agomir-140-3p NC and antagomir-140-3p NC groups (Fig. 2c, d). Conversely, the silencing of miR-140-3p expression got the opposite result.

The results of qRT-PCR (Fig. 2e) presented that the expression of ZAK in the agomir-140-3p group was lower than that in the agomir-140-3p NC group (*P* < 0.05). On the contrary, the expression of ZAK (*P* < 0.05) was increased in the antagomir-140-3p NC group. There was no significant difference in ZAK expression among the control, agomir-140-3p NC and antagomir-140-3p NC groups (*P* > 0.05). Western blot results presented (Fig. 2f–h) that the expression of ZAK (*P* < 0.05) and p-NFκB-p65 (*P* < 0.01) in the agomir-140-3p group was decreased compared with the agomir-140-3p NC group; while the expression of non-phosphorylated NF*κ*B-p65 was not significantly different (*P* > 0.05) compared with the agomir-140-3p NC group. On the contrary, the expression of ZAK and p-NFκB-p65 was increased in the antagomir-140-3p group (*P* < 0.05) compared with the antagomir-140-3p NC group; whereas the expression of non-phosphorylated NF*κ*B-p65 was not significantly different (*P* > 0.05) compared with the antagomir-140-3p NC group. The expression of ZAK, phosphorylated and non-phosphorylated NF*κ*B-p65 was not significantly different among the control, agomir-140-3p NC, and antagomir-140-3p NC groups (*P* > 0.05). Further, Western bolt was used to detect the effect of miR-140-3p on the expression of TJ-associated proteins in GECs of BTB. Results as shown in Fig. 2i, protein expression of ZO-1, occludin, and claudin-5 was increased in the agomir-140-3p group compared with the agomir-140-3p NC group. Compared with the antagomir-140-3p NC group, the decreased expression of ZO-1, occludin, and claudin-5 in the antagomir-140-3p group was observed. Immunofluorescence indicated similar results, the distribution of ZO-1, occludin, and claudin-5 was increased in the agomir-140-3p group with changing from a relatively discontinuous location to a continuous location on the cell boundary. The decreased expression was detected in the antagomir-140-3p group and their distribution changed from a relatively continuous state to a discontinuous state on the cell membrane (Fig. 2j).

### ZAK was upregulated in GECs, and ZAK promoted BTB permeability by phosphorylation of NF*κ*B-p65

**Fig. 3:**
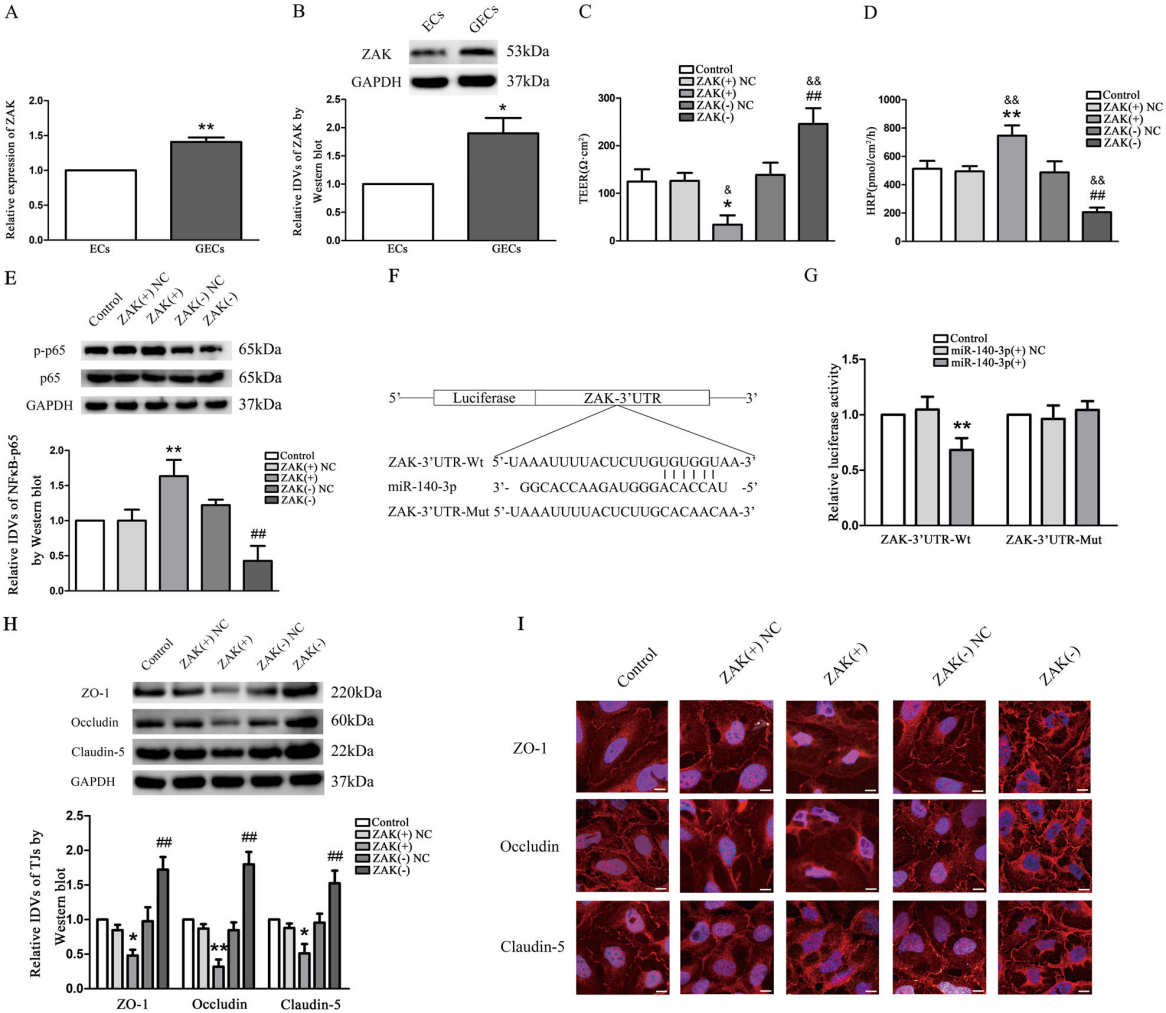
The endogenous expression of ZAK and effect of ZAK on BTB permeability were detected in GECs of BTB, and ZAK was direct target gene of miR-140-3p. **a**, **b** Relative expression of ZAK in ECs and GECs was detected by qRT-PCR and western blot. GAPDH was used as an endogenous control. Data represent mean ± SD (*n* = 3, each group). *P < 0.05 and **P < 0.01 vs. ECs group. **c** TEER values of GECs were analyzed after ZAK overexpression and silencing. Data represent means ± SD (*n* = 3, each group). *P < 0.05 vs. ZAK(+) NC group. ^##^*P* < 0.01 vs. ZAK(−) NC group. ^&^*P* < 0.05 and ^&&^*P* < 0.01 vs. Control. **d** HRP flux was performed after ZAK overexpression and silencing. Data represent means ± SD (*n* = 3, each group). **P < 0.01 vs. ZAK(+) NC group. ^##^*P* < 0.01 vs. ZAK(−) NC group. ^&&^*P* < 0.01 vs. Control. **e** The protein expression of NF*κ*B-p65 was shown by western blot. The IDVs of NF*κ*B-p65 were shown using GAPDH as an endogenous control. Data represent mean ± SD (*n* = 3, each group*)*. *P < 0.05 vs. ZAK(+) NC group. ^##^*P* < 0.01 vs. ZAK(−) NC group. **f**, **g** Relative luciferase activity was performed by dual-luciferase reporter assay. Relative luciferase activity was expressed as firefly/renilla luciferase activity. Data represent mean ± SD (*n* = 3, each group). *P < 0.05 vs. miR-140-3p NC+ ZAK wt group. **h** The protein expression of TJ-associated proteins in GECs was detected after ZAK overexpression and silencing. The IDVs of protein expression levels were shown using GAPDH as an endogenous control. Data represent mean ± SD (*n* = 3, each group). *P < 0.05 and **P < 0.01 vs. ZAK(+) NC group, ^##^*P* < 0.01 vs. ZAK(−) NC group. **i** Distribution and expression of ZO-1, occludin, and claudin-5 in GECs as detected with immunofluorescence assay. ZO-1 (red), occludin (red), and claudin-5 (red) were, respectively, labeled with fluorescent secondary antibody and nuclei were labeled with DAPI. Images were representative of five independent experiments. Scale bar = 20 µm.

Real-time PCR and Western blot were used to detect the expressions of ZAK in GECs. The results presented that the expression of ZAK in GECs was increased compared with the ECs group (Fig. 3a, b). In order to further explore the possible effect of ZAK on regulating BTB permeability, we constructed two kinds of GECs by means of ZAK(+) or ZAK(−) plasmids, and established an in vitro BTB model to detect TEER value and HRP seepage amount. The results showed that the TEER value in the ZAK(+) group was decreased (Fig. 3c) compared with the control and ZAK(+) NC groups, while the HRP seepage amount was increased (Fig. 3d) compared with the control and ZAK(+) NC groups. The ZAK(−) group had the opposite result.

Western blotting results delineated that the protein expression level of p-NFκB-p65 in the ZAK(+) group increased (*P* < 0.05) compared with the ZAK(+) NC group, while the expression of non-phosphorylated NF*κ*B-p65 showed no significant difference (*P* > 0.05) compared with the ZAK(+) NC group. On the contrary, the expression of p-NFκB-p65 in the ZAK(−) group was decreased (*P* < 0.01) compared with the ZAK(−) NC group, while the expression of non-phosphorylated NF*κ*B-p65 was not significantly different (*P* > 0.05) compared with the ZAK(−) NC group. There were no significant differences among the control, ZAK(+) NC and ZAK(−) NC groups (*P* > 0.05) (Fig. 3e).

ZAK was direct target gene of miR-140-3p by analysis of Luciferase assay. In line with expectations, the relative luciferase activity of the ZAK-3′-UTR-WT + miR-140-3p(+) group was significantly decreased in comparison with that of the ZAK-3′-UTR-WT + miR-140-3p(+) NC group (Fig. 3f, g). These results suggested that miR-140-3p inhibited the 3′-UTR function of ZAK. To further verify whether miR-140-3p directly targeted ZAK through the predicted binding site, luciferase reporter vector containing the Mut-type 3′-UTR of ZAK (ZAK-3′UTR-Mut) was constructed. Co-transfection of miR-140-3p and ZAK-3′UTR-Mut did not change luciferase activity (Fig. 3f, g). An inverse relationship was present between the miR-140-3p and ZAK expression. These results presented that ZAK was direct target of miR-140-3p with the specific binding site being located at the seed sequence.

As shown in Fig. 3h, the protein expression of ZO-1, occludin and claudin-5 in ZAK(+) group were significantly decreased compared with ZAK(+) NC group. Immunofluorescence analysis revealed that ZAK overexpression attenuated cellular membrane localization of ZO-1, occludin, and claudin-5 in GECs of the BTB (Fig. 3i), with similar changes being observed in the alteration of ZO-1, occludin, and claudin-5 protein expressions by western blotting (Fig. 3h). ZAK knockdown had the opposite effect.

### MIAT functioned as a miR-140-3p ceRNA to regulate the expression of its target gene, ZAK

**Fig. 4:**
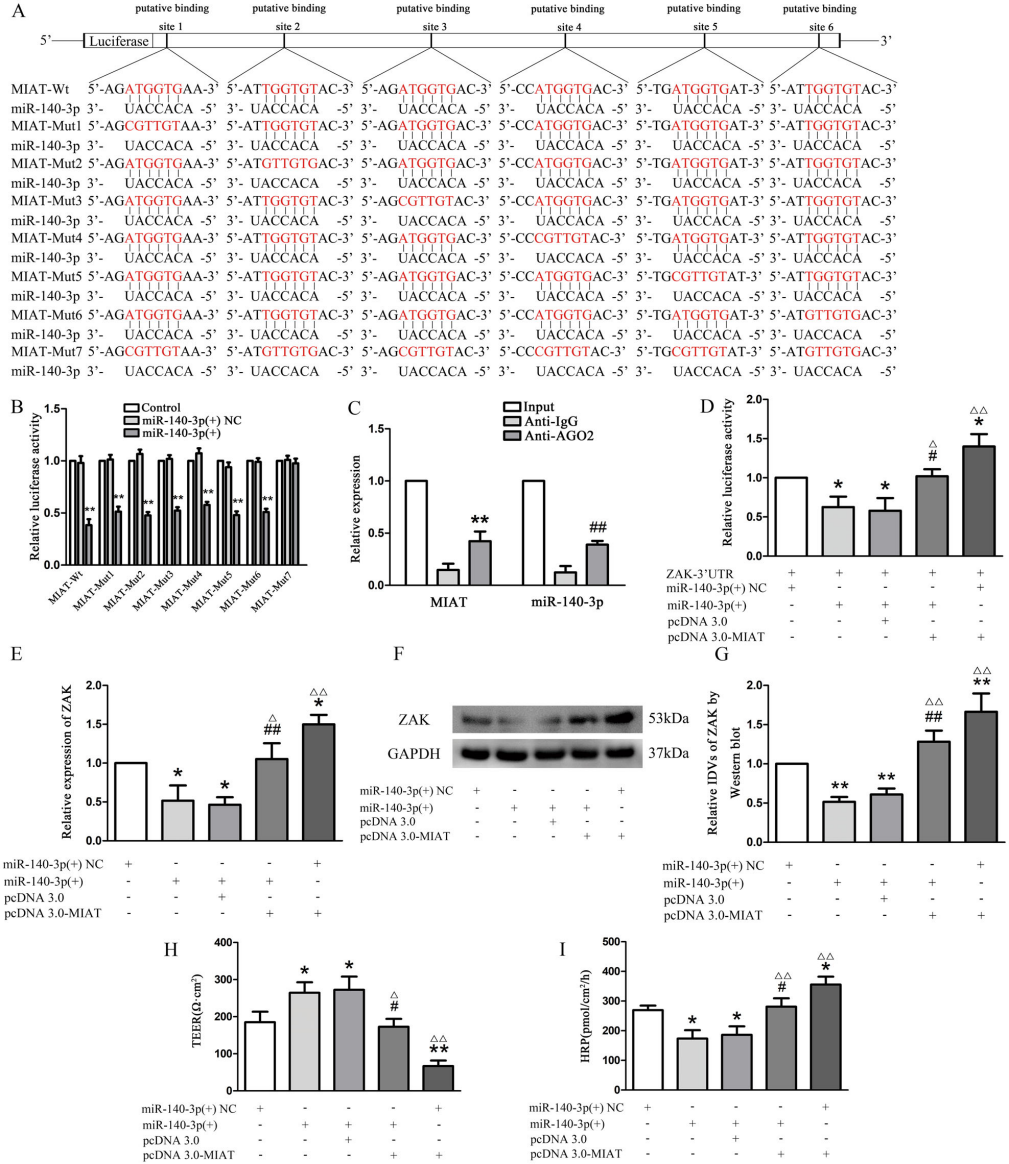
MIAT as a miR-140-3p sponge regulated the ZAK expression. **a**, **b** Relative luciferase activity was performed by dual-luciferase reporter assay. Relative luciferase activity was expressed as firefly/renilla luciferase activity. Data represent mean ± SD (*n* = 3, each group). **P < 0.01 vs. miR-140-3p(+) NC. **c** MiR-140-3p was identified in MIAT-RISC complex. Relative expression of MIAT and miR-140-3p were measured using qRT-PCR. Data represent mean ± SD (*n* = 3, each group). **P < 0.01 vs. anti-normal IgG group; ^##^*P* < 0.01 vs. anti-Ago2 in control group. **d** RLuc-ZAK-WT and miR-140-3p were co-transfected into HEK-293T cells with MIAT vector to verify the competing endogenous RNA activity of MIAT. Histogram indicated the data of luciferase activity measured 48 h after transfection. Data represent mean ± SD (*n* = 3, each group). *P < 0.05 vs. miR-140-3p(+) NC group. ^#^*P* < 0.05 vs. pcDNA 3.0+miR-140-3p(+) groups. ^△^*P* < 0.05 and ^△△^*P* < 0.01 vs. miR-140-3p(+) group. **e** 293T cells were transfected with different combinations of MIAT and agomiR-140-3p. QRT-PCR was conducted to detect ZAK expression. Data represent mean ± SD (*n* = 3, each group). *P < 0.05 vs. miR-140-3p(+) NC group. ^##^*P* < 0.01 vs. pcDNA 3.0+miR-140-3p(+) groups. ^△^*P* < 0.05 and ^△△^*P* < 0.01 vs. miR-140-3p(+) group. **f**, **g** The protein expression of ZAK was shown by western blot. The IDVs of ZAK were shown using GAPDH as an endogenous control. Data represent mean ± SD (*n* = 3, each group). **P < 0.01 vs. miR-140-3p(+) NC group. ^##^*P* < 0.01 vs. pcDNA3.0+miR-140-3p(+) groups. ^△△^*P* < 0.01 vs. miR-140-3p(+) group. **h** MIAT and miR-140-3p(+) changed TEER values of GECs. Data represent mean ± SD (*n* = 3, each group). *P < 0.05 and **P < 0.01 vs. miR-140-3p(+) NC group. ^#^*P* < 0.05 vs. pcDNA3.0+miR-140-3p(+) groups. ^△^*P* < 0.05 and ^△△^*P* < 0.01 vs. miR-140-3p(+) group. **i** MIAT and miR-140-3p(+) changed HRP flux of GECs. Data represent mean ± SD (*n* = 3, each group). *P < 0.05 vs. miR-140-3p(+) NC group. ^#^*P* < 0.05 vs. pcDNA3.0+miR-140-3p(+) groups. ^△△^*P* < 0.01 vs. miR-140-3p(+) group.

Using BioEdit, we predicted six miR-binding sites on the MIAT sequence and suggested that MIAT might function as a molecular sponge of miR-140-3p to regulate the BTB permeability. The result of the dual-luciferase reporter assay was consistent with our expectation. Compared with the control group, the miR-140-3p(+) and MIAT-Wt/-Mut1/-Mut2/-Mut3/-Mut4/-Mut5/-Mut6 double transfection group showed lower luciferase activity. However, there was no significant change in the luciferase activity between the miR-140-3p(+) and MIAT-Mut7 double transfection group and the control group (Fig. 4a, b). RNA induced silencing complexes are formed by miRNA ribonucleoprotein complexes (miRNPs), which is present in anti-Ago2 immunoprecipitates. Therefore, anti-Ago2 immunoprecipitates contain miRNAs and their interacting RNA components. RIP assay was performed using anti-AGO2 in the GECs extract, and it was found that MIAT and miR-140-3p were enriched preferentially in miRNPs containing AGO2 compared with anti-IgG immunoprecipitates (Fig. 4c). Combining the two results, MIAT functioned as a miR-140-3p ceRNA to regulate the expression of ZAK. To further reveal the molecular mechanism of MIAT functioned as a competing endogenous RNA to competitively regulate the expression of ZAK, ZAK 3′-UTR was inserted into luciferase coding region (RLuc-ZAK-wt) and transfected into HEK-293T cells with agomir-140-3p or MIAT. MIAT cDNA was cloned into pcDNA3.0 vector and transfected into 293T cells with different miR-140-3p and ZAK vectors. Luciferase assay indicated that agomir-140-3p significantly reduced RLuc-ZAK-wt luciferase activity, while MIAT overexpression partially rescued miR-140-3p mediated the inhibition of RLuc-ZAK-wt luciferase activity, suggesting that MIAT had cross-talk through competing miR-140-3p binding (Fig. 4d).

The results of qRT-PCR and Western blot (Fig. 4e–g) presented that miR-140-3p overexpression significantly reduced ZAK level (*P* < 0.05) compared with the agomir-140-3p NC group, while the decreased ZAK level partly recovered after MIAT overexpression (*P* < 0.01). MIAT overexpression significantly increased ZAK level (*P* < 0.05) compared with the pcDNA 3.0 + miR-140-3p(+) groups. These results suggest that there is interplay among MIAT, miR-140-3p, and ZAK. After an in vitro BTB model establishment, it was found (Fig. 4h, i) that the TEER value of the miR-140-3p overexpression group was increased (*P* < 0.05) compared with the agomir-140-3p NC group, and the HRP leakage amount was decreased (*P* < 0.05). When MIAT level increased, TEER value and HRP leakage partly recovered. In other words, TEER value decreased (*P* < 0.01), and HRP exudate increased (*P* < 0.05) in pcDNA3.0-MIAT + miR-140-3p(+) groups compared with the pcDNA3.0 + miR-140-3p(+) groups.

### NF*κ*B-p65 was upregulated in GECs, and NF*κ*B-p65 knockdown reduced the permeability of BTB

**Fig. 5:**
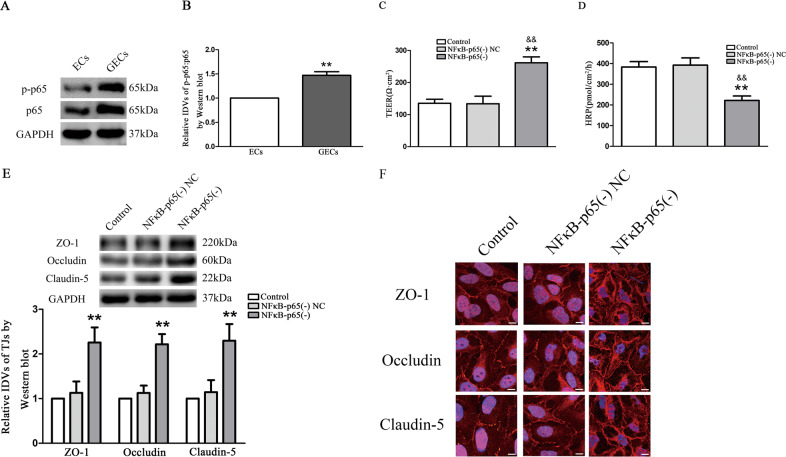
The endogenous expression of NF*κ*B-p65 was detected in GECs of BTB, and NF*κ*B-p65 knockdown reduced BTB permeability. **a**, **b** Relative expression of NF*κ*B-p65 in ECs and GECs was detected by Western blot. The IDVs was shown using GAPDH as an endogenous control. Data represent mean ± SD (*n* = 3, each group). **P < 0.01 vs. ECs group. **c** TEER values of GECs were detected after NF*κ*B-p65 silencing. Data represent means ± SD (*n* = 3, each group). **P < 0.01 vs. NF*κ*B-p65(−) NC group. ^&&^*P* < 0.01 vs. Control. **d** HRP flux was performed after NF*κ*B-p65 knockdown. Data represent means ± SD (*n* = 3, each group). **P < 0.01 vs. NF*κ*B-p65(−) NC group. ^&&^*P* < 0.01 vs. Control. **e** The TJ-associated proteins expressions in GECs were d**e**tected after NF*κ*B-p65 silencing. The IDVs of protein expression levels were shown using GAPDH as an endogenous control. Data represent mean ± SD (*n* = 3, each group). **P < 0.01 vs. NF*κ*B-p65(−) NC group. **f** Immunofluorescent localization of ZO-1, occludin, and claudin-5 in GECs. ZO-1 (red), occludin (red), and claudin-5 (red) were, respectively, labeled with fluorescent secondary antibody and nuclei were labeled with DAPI. Images were representative of five independent experiments. Scale bar = 20 µm.

Western blot were used to detect the protein expression of NF*κ*B-p65 in GECs. The results affirmed that NF*κ*B-p65 expression in GECs group was increased compared with the ECs group (Fig. 5a, b). In order to further investigate the function of NF*κ*B-p65 in regulating the permeability of BTB, we constructed the GECs of NF*κ*B-p65 knockdown and established an in vitro BTB model to facilitate detection of the TEER value and HRP exudation amount. It was found that TEER value increased (Fig. 5c) and HRP exudation decreased (Fig. 5d) in the NF*κ*B-p65(−) group compared with the NF*κ*B-p65(−) NC group. As shown in Fig. 5e, protein expression of ZO-1, occludin, and claudin-5 in NF*κ*B-p65(−) group was significantly increased compared with the NF*κ*B-p65(−) NC group. Sh-NF*κ*B-p65 enhanced cellular membrane localization of ZO-1, occludin, and claudin-5 in GECs of the BTB by immunofluorescence (Fig. 5f).

### NF*κ*B-p65 as a transcription repressor inhibited the expression of TJ-associated proteins by binding their promoter regions

**Fig. 6:**
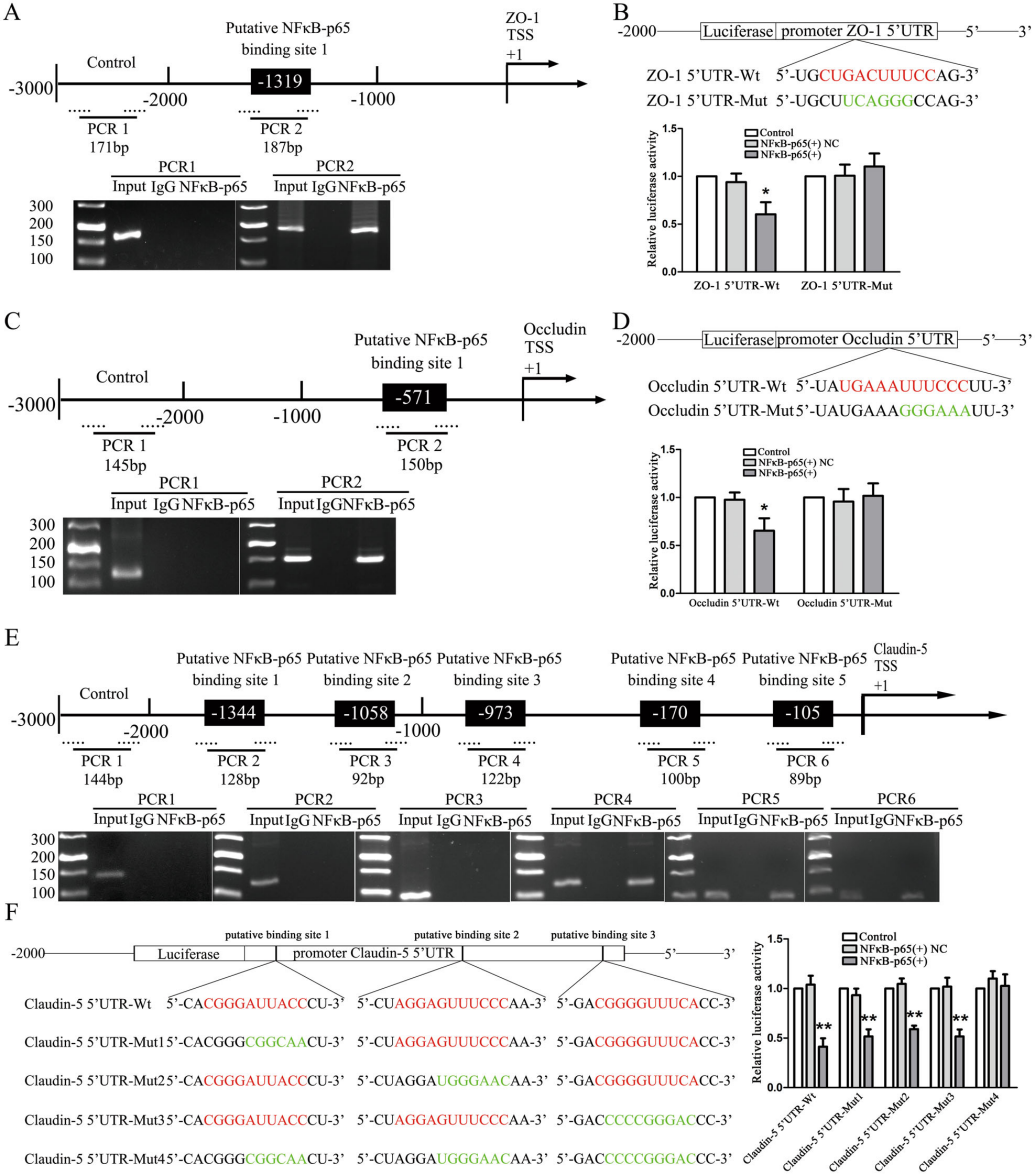
NF*κ*B-p65 bound to the promoters of TJ-associated proteins in GECs of BTB. **a** Schematic representation of the human ZO-1 promoter regions in 3000 bp upstream of the transcription start site (TSS, designated as +1). Chip PCR products for binding sites and upstream regions not expected to associate with NF*κ*B-p65 were amplified by PCR using their specific primers. Images were representative of independent Chip experiments. **b** Dual luciferase reporter assays were performed to determine the binding sites of NF*κ*B-p65 and ZO-1 in HEK-293T cells. Data represent mean ± SD (*n* = 3, each). *p < 0.05 vs. NF*κ*B-p65(+) NC group. **c** Schemati**c** representation of the human occludin promoter regions in 3000 bp upstream of the transcription start site (TSS, designated as +1). Chip PCR products for binding sites and upstream regions not expected to associate with NF*κ*B-p65 were amplified by PCR using their specific primers. Images were representative of independent Chip experiments. **d** Dual luciferase reporter assays were performed to determine the binding sites of NF*κ*B-p65 and occludin in HEK-293T cells. Data represent mean ± SD (*n* = 3, each). *p < 0.05 vs. NF*κ*B-p65(+) NC group. **e** Schematic representation of the human claudin-5 promoter regions in 3000 bp upstream of the transcription start site (TSS, designated as +1). Chip PCR products for binding sites and upstream regions not expected to associate with NF*κ*B-p65 were amplified by PCR using their specific primers. Images were representative of independent Chip experiments. **f** The putative NF*κ*B-p65 binding sites in the claudin-5 and designed mutant sequences are presented. Relative luciferase activity was performed by dual-luciferase reporter assays. Data represented as mean ± SD (*n* = 3). **P < 0.01 vs. NF*κ*B-p65(+) NC group.

NF*κ*B-p65 could specifically bind to the promoter region of target gene and regulated gene expression. Application of JASPAR database to predict the binding sites of NF*κ*B-p65 in the promoter region of ZO-1, occludin, and claudin-5, suggested that the promoter sequence of ZO-1, occludin, and claudin-5 contained 1, 1, and 5 potential binding sites of NF*κ*B-p65 (Fig. S2c–e). PCR primers were designed including predicted sites and negative control primers were random designed in 3000 bp promoter region. Figure 6a, c, e depicted that IgG could not precipitate the sequence of ZO-1, occludin, and claudin-5 promoters. Their negative control areas showed no fundraising of NF*κ*B-p65. In addition, among the predicted binding sites, NF*κ*B-p65 was recruited to ZO-1 binding site 1, occludin binding site 1, and claudin-5 binding site 1, 2, and 3.

Next, a dual-luciferase reporter assay was performed to confirm the putative binding sites of NF*κ*B-p65 in the ZO-1, occludin and claudin-5 5′-UTR. As shown in Fig. 6b, d, luciferase activity was lower in the ZO-1 and occludin 5′-UTR-Wt+NF*κ*B-p65(+) group than in the ZO-1 and occludin 5′-UTR-Wt+NF*κ*B-p65(+) NC group. However, luciferase activity remained similar levels in HEK-293T cells co-transfected with NF*κ*B-p65(+) and a reporter vector containing a correspondingly mutated NF*κ*B-p65 binding fragment of ZO-1 and occludin.

Subsequently, luciferase activity was decreased in HEK-293T cells in the with NF*κ*B-p65(+) and claudin-5 mRNA 5′-UTR-Wt, NF*κ*B-p65(+) and claudin-5 5′-UTR-Mut1, NF*κ*B-p65(+) and claudin-5 5′-UTR-Mut2, as well as NF*κ*B-p65(+) and claudin-5 5′-UTR-Mut3 groups in Fig. 6f. However, luciferase activity kept the original levels in HEK-293T cells with co-transfected into NF*κ*B-p65(+) and containing the combined mutated NF*κ*B-p65-binding sites vector. The above data suggested that NF*κ*B-p65 inhibited claudin-5 transcription by targeting the aforementioned three sites in the claudin-5 5′-UTR. These results confirmed that NF*κ*B-p65 bound to and inhibited the expression of ZO-1, occludin, and claudin-5 promoter regions.

### Combined treatment of MIAT and miR-140-3p promoted Dox delivery across BTB to induce apoptosis of glioma cells

**Fig. 7:**
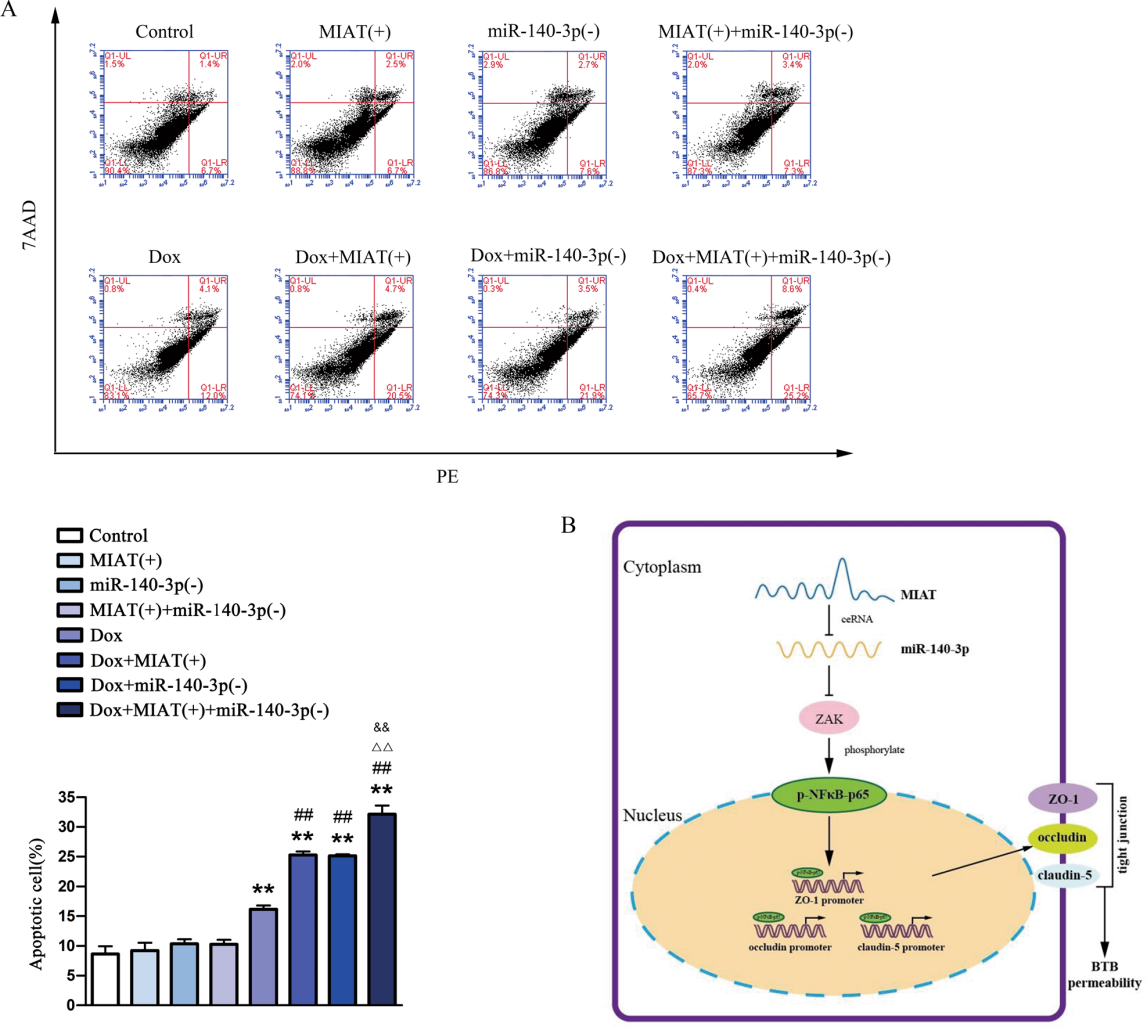
MIAT and miR-140-3p enhanced the effect of Dox on promoting apoptosis of U87 cells. **a** Apoptosis analysis in U87 cells was evaluated by Annexin V-PE/7-AAD staining. Data represent mean ± SD (*n* = 3, each). **P < 0.01 vs. control group; ^##^*P* < 0.01 vs. Dox group. ^△△^*P* < 0.01 vs. Dox+MIAT(+) group; ^&&^*P* < 0.01 vs. Dox+miR-140-3p(−) group. **b** Schematic diagram of MIAT/miR-140-3p/ZAK/NF*κ*B-p65 pathway to regulate BTB permeability.

As shown in Fig. 7a, compared with the Dox group, the apoptosis rate of U87 cells was significantly higher in the MIAT(+) + Dox group, miR-140-3p(−) + Dox group, MIAT(+) + miR-140-3p(−) + Dox group. Moreover, compared with MIAT(+) or miR-140-3p(−) single treated with Dox group, the apoptosis rate of U87 cells was increased markedly in the double of MIAT(+) and miR-140-3p(−) genes in GECs treated with Dox group.

## Discussion

In this study, we demonstrated that MIAT was highly expressed in GECs, and the increased expression of MIAT enhanced the permeability of BTB. We provided direct evidence that MIAT was involved in modulating BTB permeability. MiR-140-3p was low expressed in GECs, and the decreased expression of miR-140-3p had a negative post-transcriptional regulation to ZAK, leading to the increased expression of ZAK. Furthermore, the high expression of MIAT played the role of ceRNA for further inhibiting the function of miR-140-3p, thereby enhancing the above effects. Moreover, the highly expressed ZAK enhanced the transcriptional repression activity of NF*κ*B-p65, which acted as a negative transcription factor to regulate the expression of TJ-associated proteins and thus to modulate the permeability of BTB. Single or combined application of MIAT and miR-140-3p with Dox significantly promoted the apoptosis of U87 cells. Here, we clarified the regulatory mechanism of MIAT regulating BTB permeability through exerting ceRNA effect on miR-140-3p. To our knowledge, this was the first report about the study on regulatory mechanism of BTB permeability using MIAT as a ceRNA.

It has reported that lncRNAs could regulate the expression of critical genes and signaling pathways during the tumourigenesis and malignant processes, working as the regulators of the cancer^26^. MIAT was expressed in the focal area of the adult brain, and abundantly expressed in the nervous system and retinal tissue^27^. However, there’s no report on the relationship between MIAT and BTB permeability. Previous studies focused on the correlation effect between MIAT, myocardial fibrosis, diabetic cardiomyopathy, and diabetic retinopathy^28–30^. Our data affirmed that MIAT was endogenously expressed in ECs of BBB, and was upregulated in GECs of BTB. Similarly, expression of MIAT was significantly higher in diabetic retina than that in non-diabetic retin^28^, which was consistent with our results. To better understand the mechanism of MIAT modulating BTB permeability, we further overexpressed and silenced MIAT in GECs to impair BTB integrity and enhance BTB permeability by downregulating TJ-associated proteins, while promoting the ZAK expression and ratio of phosphorylation to non-phosphorylation of NF*κ*B (p-p65:p65) in GECs. Consistent with our results, Jiang et al.^29^ found that MIAT regulated endothelial cells function and changed the expression of TJ-associated proteins. These findings indicated that TJ function of ECs was influenced by the expression of MIAT.

More and more studies confirmed that miRNA was involved in regulating the BTB permeability. For instance, miR-138-5p and miR-150-5p’s contribution to an increase in BTB permeability was associated with TJ disassembly as achieved by directly targeting FOSL2^13^. MiR-18a participated in the regulation of vascular function and improved the function of ECs in human brain with arteriovenous malformations. Moreover, overexpression of miR-18a impaired the integrity of BTB and significantly increased the permeability of BTB by reducing ZO-1, occludin, and claudin-5 expression^30^. In addition, previous experiments demonstrated that decreased expression of ZO-1, occludin, and claudin-5 increased BTB permeability^31^. Our study demonstrated that the expression of miR-140-3p was suppressed in GECs, and some literature reported that miR-140-3p was downregulated in many tissue cancers^12^, and its expression in lung cancer cells was lower than that in normal lung epithelial cells^32^, which was consistent with our results. In addition to regulating the expression of genes related to the nervous system, miR-140-3p can also regulate the other target genes expression^33^. MiR-140-3p in ECs could reduce the ability of tubule formation by negatively regulating ECs proliferation, migration, and invasion in diabetic vascular disease through direct targeting FOXK2 signal conduction^34^. Flamini VE reported that miR-140-3p mimics suppressed the invasion of lung cancer cells and the inhibitory effect of the miR-140-3p on adhesion through downregulating F-1, c-Jun, and other AP-1 transcription factors expression^32^. Similary, our results illustrated that overexpression or silencing of miR-140-3p in GECs reduced or increased BTB permeability, demonstrating that miR-140-3p was involved in the regulation of BTB permeability. Double luciferase reporter assay showed that miR-140-3p directly targeted ZAK 3′-UTR and suppressed ZAK expression, suggesting that miR-140-3p negatively regulated ZAK expression and modulated BTB permeability. The above results indicated that these gene variations in expression might be due to miR-140-3p exerting a different role in different type of cells. When collating these findings with our current results, it appeared that changes of miR-140-3p expression might play a key role in regulating BTB permeability.

ZAK, a sterile alpha motif and leucine zipper containing kinase belonging to a member of the MAPKKK family, was involved in critical biological processes including cell growth, differentiation, cytoskeletal changes, and gene expression^35^. In the last decades, researches about ZAK were mainly focused on its role in the regulation of cell growth. Tang reported that ZAK expression had negative correlation with breast cancer survival^36^, and some studies confirmed that ZAK was up-regulated in gastric, breast, bladder, and colorectal cancers^37^. Here, the results in this work revealed that the ZAK expression in GECs was significantly increased compared with that in ECs. Recently, ZAK had been shown to play important roles in cell cycle, apoptosis, tumor cell transformation, as well as in a variety of physiological and pathological processes^38^. Our results confirmed that overexpression of ZAK increased BTB permeability and downregulated expression of ZO-1, occludin, and claudin-5, whereas ZAK silencing had the opposite results. All the above experimental results displayed that ZAK as a key molecular modulated the permeability of GECs of BTB.

CeRNA hypothesized that lncRNAs, circle RNA, and transcribed pseudogenes could be used as molecular sponges of miRNA to compete for the binding of normal miRNA molecules through miRNA recognition elements^36–39^. A better understanding of RNA cross-talk would provide novel insights into the mechanisms of gene regulatory networks in some diseases, which might be valuable for developing novel diagnostic markers and targeted therapy. Our study demonstrated that MIAT as a miR-140-3p sponge might regulate ZAK expression and BTB permeability. Similarly, Luan et al. reported that MIAT also functioned as a ceRNA to inhibit proliferation, migration and invasion, as well as promoting apoptosis in breast cancer cells through binding to miR-155-5p^40^. In addition, Fu confirmed that MIAT exerted a ceRNA role to inhibit the function of miR-29b and weak its activity against multiple myeloma^41^. Taken together, These studies suggested that MIAT might regulate the function of related miRNAs through ceRNA, thereby affecting the expression of downstream related genes and disease progression.

NF*κ*B was a predominate on REDOX sensitive transcription factor. Its activation was an initial signaling events, followed by activating the other inflammatory pathways, which led to the microvascular dysfunction and type 2 diabetes mediated injury caused by affecting inflammation, cell proliferation, angiogenesis, and cell adhesion gene expression^42^. Our results showed that NF*κ*B-p65 was upregulated in GECs of BTB, and NF*κ*B-p65 knockdown decreased BTB permeability. Similarly, Liu found that the activation of NF*κ*B-p65 induced by rhEPO might be involved rhEPO-mediated the opening of BBB^25^. It has been reported that overexpression of ZAK promoted the activity of AP1, NF*κ*B and β-catenin protein^43^, and ZAK exerted a critical function including TGF-*β*-dependent SMAD2/3 phosphorylation, target gene expression, and aggressive cell phenotypic transformation^17^. Our results showed that over-expression of ZAK resulted in phosphorylation of transcription factors NF*κ*B(p65). Moreover, results which were opposite to that obtained with the ZAK silencing group. It was reported that ZAK could also phosphorylate other transcription factors^15^. Overexpression of ZAK triggered the p38 and JNK pathway and also activated the expression and nuclear translocation of p-GATA4 and p-c-Jun transcription factors^15^. Accordingly, these studies lent credence to the possibility that ZAK could phosphorylate different transcription factors under different physiological and pathological conditions. NF*κ*B-p65 inhibited the expression of E-cadherin and ZO-1 in breast epithelial cells, and regulated its target genes transcription by binding to the promoter region of target genes^44,45^. The production of TNF-*α* in some pathophysiological process disrupted the barrier function by reducing the levels of the TJ-associated protein through NF*κ*B signaling pathway^46^. Ser536 was located at the COOH terminal TAD of NF*κ*B-p65, and its phosphorylation by TNF-*α* etc played a key role in the transcriptional activation^44^. Our results confirmed that phosphorylation of NF*κ*B-p65 increased in GECs, and phosphorylation of NF*κ*B-p65 at ser536 decreased the promoter activity of ZO-1, occludin, and claudin-5, which resulted to an increase in BTB permeability. Consistent with our findings, Aslam reported that over-expression of the NF*κ*B subunit p65 alone repressed claudin-5 promoter activity in mouse brain endothelial cells^47^. Taken together, the data above indicated that ZAK might facilitate BTB permeability by repressing the expression of TJ-associated protein through phosphorylation of NF*κ*B-p65 at ser536.

Dox is considered one of the most effective chemotherapeutic agents, used as a first-line anticancer for hematologic and solid tumors. In the treatment of glioma, due to the presence of the BTB, it is difficult for Dox to enter the brain and achieve effective therapeutic concentration. While lncRNA MIAT and miRNA can effectively promote Dox through BTB to induce glioma cell apoptosis. We found that over-expression of MIAT and silencing of miR-140-3p alone or in combination significantly increased apoptosis of U87 cells by Dox. The results indicated that the MIAT and miR-140-3p alone or in combination could enhance the antitumor effect of Dox by promoting its penetrating capability across BTB.

In summary, our data suggested that upregulated MIAT acted as a miR-140-3p ceRNA to elevate the ZAK expression. ZAK’s contribution to phosphorylation of NF*κ*B-p65 was associated with downregulated TJ-associated expression and an increase in BTB permeability. The combined application of MIAT and miR-140-3p promoted Dox delivery through BTB, thereby inducing apoptosis of glioma cells. These studies could provided new therapeutic strategies for glioma.

## Supplementary information

Supplementary Fig. S1.

Supplementary Fig. S2

Supplementary fig legend

Supplementary table1

Supplementary table2

Supplementary table3

Supplementary table4

## Data Availability

The data supporting the conclusion of this research has been included in this published article and its Supplementary files.
