# Supplementary figures and images for: Long non-coding RNA MIAT regulates blood tumor barrier permeability by functioning as a competing endogenous RNA
Source: Cell Death Dis. 2020 Oct 30;11(10):936. doi: 10.1038/s41419-020-03134-0 (PMC7603350; doi:10.1038/s41419-020-03134-0)

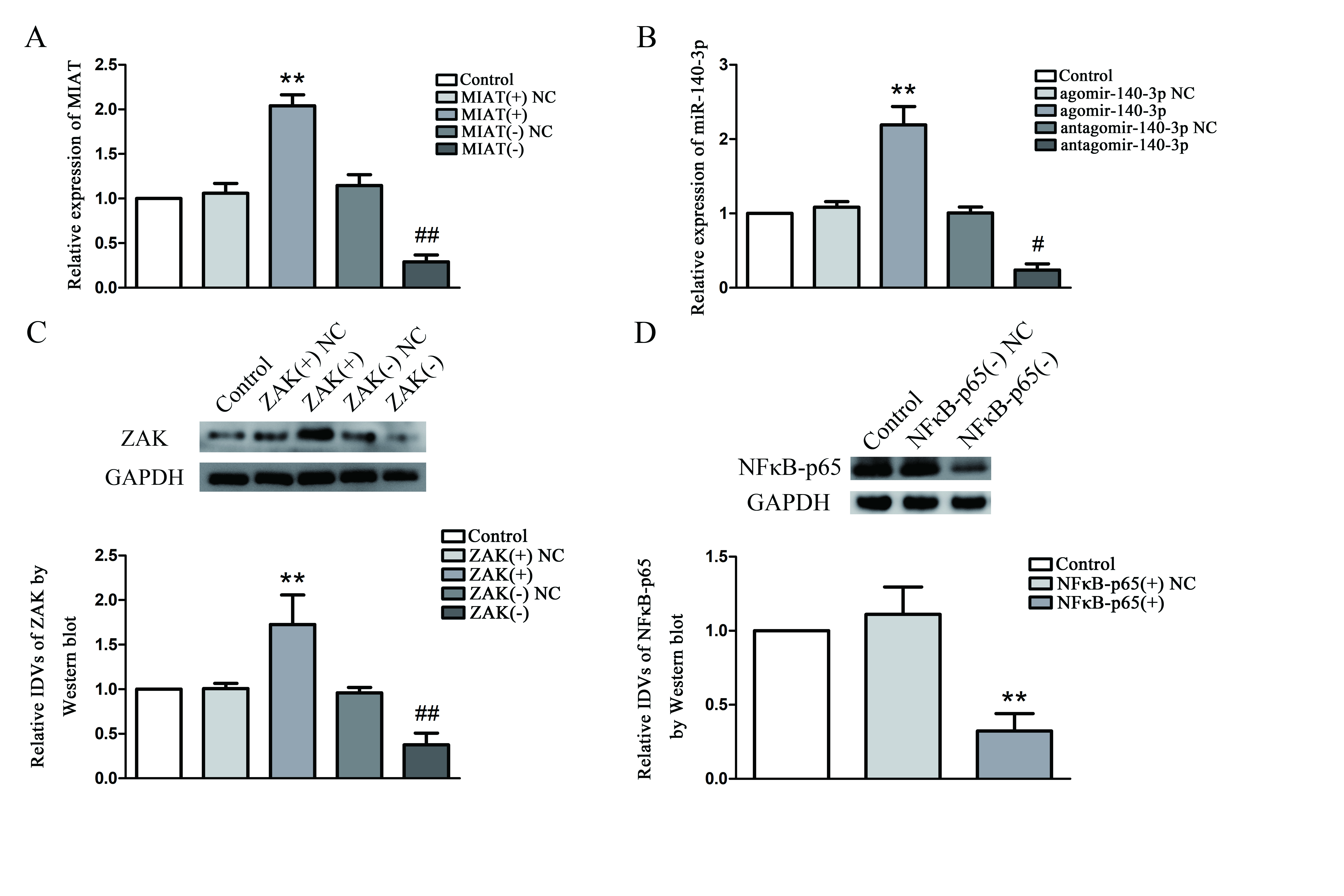

Supplement: Supplementary file 1 — Supplementary Fig. S1. [file 41419_2020_3134_MOESM1_ESM.jpg]

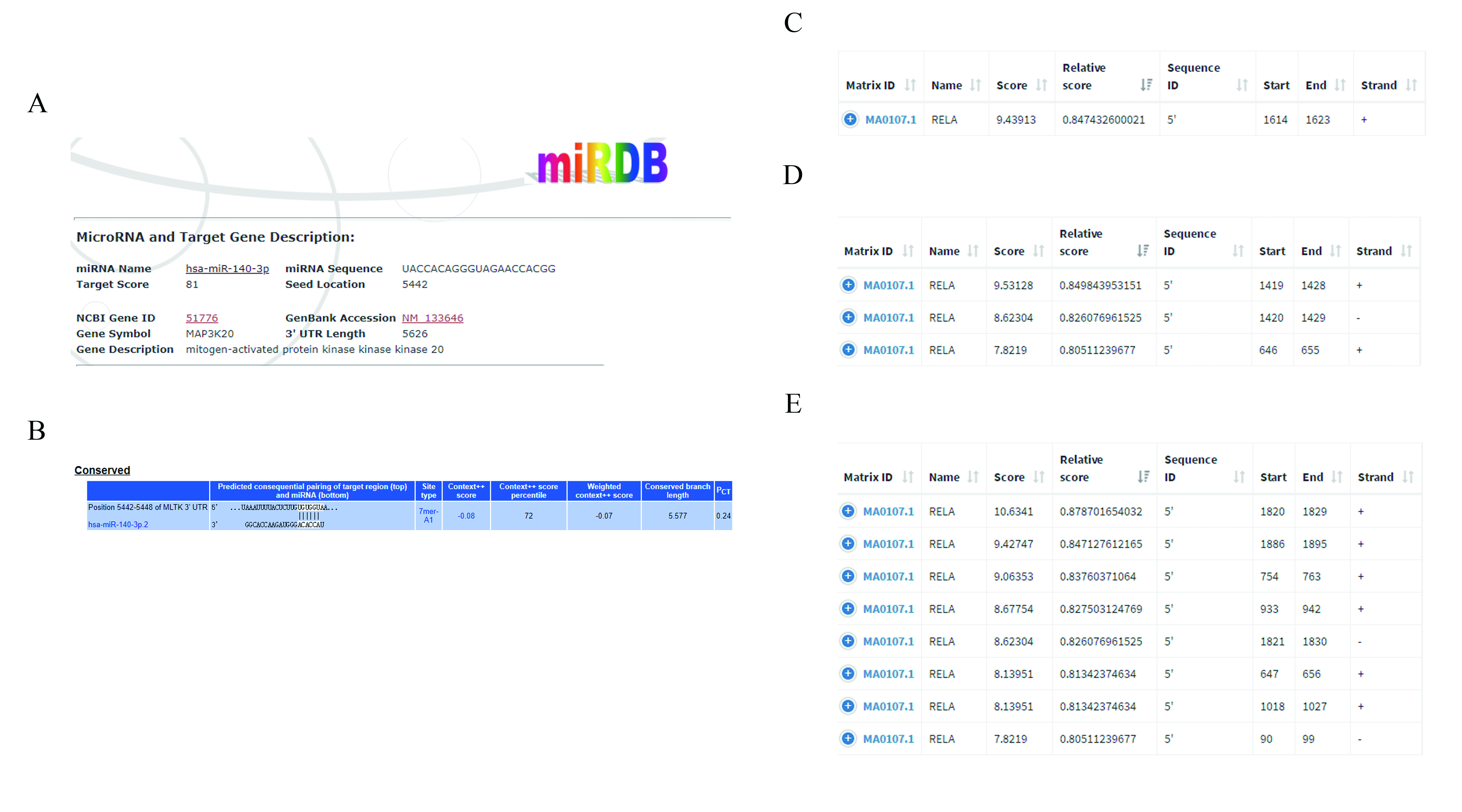

Supplement: Supplementary file 2 — Supplementary Fig. S2 [file 41419_2020_3134_MOESM2_ESM.jpg]
