# Supplementary material for: Long non-coding RNA MIAT regulates blood tumor barrier permeability by functioning as a competing endogenous RNA
Source: Cell Death Dis. 2020 Oct 30;11(10):936. doi: 10.1038/s41419-020-03134-0 (PMC7603350; doi:10.1038/s41419-020-03134-0)
Supplement: Supplementary file 3 — Supplementary fig legend [file 41419_2020_3134_MOESM3_ESM.docx]

**Supplementary Information**

**Supplementary Fig. S1.** **A.** The transfection efficiency of MIAT was detected by qRT-PCR. Data represent mean ± SD (n = 3, each group). ***P* < 0.01 versus MIAT(+) NC group; ^##^*P* < 0.01 versus MIAT(-) NC group. **B**. The transfection efficiency of miR-140-3p was detected by qRT-PCR. Data represent mean ± SD (n = 3, each group). ***P* < 0.01 versus agomir-140-3p NC group; ^#^*P* < 0.05 versus antagomir-140-3p NC group. **C.** The transfection efficiency of ZAK was detected by western blot. Data represent mean ± SD (n = 3, each group). ***P* < 0.01 versus ZAK(+) NC group; ^##^*P* < 0.01 versus ZAK(-) NC group. **D.** The silent transfection efficiency of NF*κ*B-p65 was detected by western blot. Data represent mean ± SD (n = 3, each group). ***P* < 0.01 versus NF*κ*B-p65(-) NC group.

**Supplementary Fig. S2.** **A**. A binding site between miR140-3p seed region and at the 3′ -UTR region of ZAK was prediced by miRDB. **B**. A binding site between miR140-3p seed region and at the 3′ -UTR region of ZAK was prediced by TargetScan7.2. **C.** The potential binding sites of p-NF*κ*B-p65 in the upstream promoter region of ZO-1 were found by JASPAR. **D.** The potential binding sites of p-NF*κ*B-p65 in the upstream promoter region of occludin were found by JASPAR. **E.** The potential binding sites of p-NF*κ*B-p65 in the upstream promoter region of claudin-5 were found by JASPAR.
