# Supplementary material for: Long non-coding RNA MIAT regulates blood tumor barrier permeability by functioning as a competing endogenous RNA
Source: Cell Death Dis. 2020 Oct 30;11(10):936. doi: 10.1038/s41419-020-03134-0 (PMC7603350; doi:10.1038/s41419-020-03134-0)
Supplement: Supplementary file 4 — Supplementary table1 [file 41419_2020_3134_MOESM4_ESM.docx]

Table 1

Primers and probes used for qRT-PCR

| Primer or Probe | Gene | Sequence(5’->3’) or Assay ID |
| --- | --- | --- |
| Primer | MIAT | F:CTTCACCTTGACTAACTCCTGCCTTC |
|  |  | R:CCAGCCATGCCGACATCCAAG |
|  | ZAK | F:GGATTCCTACGCTGCTGCTGTG |
|  |  | R:GACGAGCTTCTGGACTGGTTCAC |
|  | NFκB-p65 | F:ACCCCTTCCAAGAAGAGCAG |
|  |  | R:AGCCTGGTCCCGTGAAATAC |
|  | GAPDH | F:AAATCCCATCACCATCTTCCAG |
|  |  | R:TGATGACCCTTTTGGCTCCC |
|  | miR-140-3p | UACCACAGGGUAGAACCACGG |
|  | U6 | (D356-03, TaKaRa, Dalian, China) |
