# Supplementary material for: Long non-coding RNA MIAT regulates blood tumor barrier permeability by functioning as a competing endogenous RNA
Source: Cell Death Dis. 2020 Oct 30;11(10):936. doi: 10.1038/s41419-020-03134-0 (PMC7603350; doi:10.1038/s41419-020-03134-0)
Supplement: Supplementary file 5 — Supplementary table2 [file 41419_2020_3134_MOESM5_ESM.docx]

Table 2

Sequences of shRNA template

| Gene | Sequence(5’->3’) | |
| --- | --- | --- |
| MIAT | Sense | GCCAATAGTTGGAGAAACT |
|  | Antisense | CATGAACGTCATGAAGTAG |
| MiR-140-3p | Sense | CCGTGGTTCTACCCTGTGGTA |
|  | Antisense | AGGCCATTCTGTTGCCGGCTT |
| ZAK | Sense | GGAAGCAGGTCCGACTCAAGT |
|  | Antisense | GCCATGGAACTTAACGGCGAG |
| NFκB-p65 | Sense | CCGGGCCTTAATAGTAGGGTAAGTTCTCGAGAACTTACCCTACTATTAAGGCTTTTT |
|  | Antisense | CCATAGGTTAACTCGTTGCTGAACCGGTTAGCTATTTAATACCGGTTGAGCCATTTA |

Sequences of preRNA template

| Gene | Sequence(5’->3’) | |
| --- | --- | --- |
| MiR-140-3p | Sense | TACCACAGGGTAGAACCACGG |
|  | Antisense | GAACCTGACCGGATAAGCCGA |
