# Supplementary material for: Long non-coding RNA MIAT regulates blood tumor barrier permeability by functioning as a competing endogenous RNA
Source: Cell Death Dis. 2020 Oct 30;11(10):936. doi: 10.1038/s41419-020-03134-0 (PMC7603350; doi:10.1038/s41419-020-03134-0)
Supplement: Supplementary file 6 — Supplementary table3 [file 41419_2020_3134_MOESM6_ESM.docx]

Table 3

Wild-type and mutant plasmid sequences

ZAK：

| Wild-type plasmid sequences | Mutant plasmid sequences |
| --- | --- |
| UAAAUUUUACUCUUGUGUGGUAA | UAAAUUUUACUCUUGCACAACAA |

ZO-1

| Wild-type plasmid sequences | Mutant plasmid sequences |
| --- | --- |
| UGCUGACUUUCCAG | UGCUUCAGGGCCAG |

Occludin

| Wild-type plasmid sequences | Mutant plasmid sequences |
| --- | --- |
| UAUGAAAUUUCCCUU | UAUGAAAGGGAAAUU |

Claudin-5

| Wild-type plasmid sequences | Mutant plasmid sequences |
| --- | --- |
| CACGGGAUUACCCU | CACGGGCGGCAACU |
| CUAGGAGUUUCCCAA | CUAGGAUGGGAACAA |
| GACGGGGUUUCACC | GACCCCCGGGACCC |
