# Supplementary material for: Long non-coding RNA MIAT regulates blood tumor barrier permeability by functioning as a competing endogenous RNA
Source: Cell Death Dis. 2020 Oct 30;11(10):936. doi: 10.1038/s41419-020-03134-0 (PMC7603350; doi:10.1038/s41419-020-03134-0)
Supplement: Supplementary file 7 — Supplementary table4 [file 41419_2020_3134_MOESM7_ESM.docx]

Table 4

Primers used for CHIP experiments

| Gene | Binding site or control | Sequence(5’->3’) | Product size(bp) | Annealing temperature(°C) |
| --- | --- | --- | --- | --- |
| ZO-1 | PCR1 | TGGTCAACAAAGATGAAAGTGCAGC | 187 | 58.14 |
|  |  | CCATTTTCCTCATTCAGTGTGCTTTGAG |  |  |
| occludin | PCR1 | AGAACTATAATTGCCACATCCTGGAGTAC | 150 | 57.86 |
|  |  | TCCGGGTGCTGTATTCTATTTCTTGATC |  |  |
| claudin-5 | PCR1 | GCCTCCTGAGTAGGTGGGATTAC | 128 | 59.48 |
|  |  | GGATCACCTCAGGTCAGGAGTTC |  |  |
|  | PCR2 | GATGGAGTGCAATGGCATGATCTC | 92 | 58.55 |
|  |  | AATCCGTTACTCGGGTAGCTGAG |  |  |
|  | PCR3 | CTCAGCTACCCGAGTAACGGATTAC | 122 | 58.8 |
|  |  | ACTTGAGGTCAGGAGTTTGAGACC |  |  |
|  | PCR4 | CTGAAAGATAGAAGAAACGCTGTTCTTCAC | 100 | 57.94 |
|  |  | ATTTAGGCTCAAGATTCCTGGGGATTTG |  |  |
|  | PCR5 | AGCCTAAATTTAGCTTATCCTTGGAAGGTC | 89 | 57.7 |
|  |  | AGAAAAGCCAAGTTTATCTTGAGCAGC |  |  |
